# Meridian-sinew physical therapy modulates regional brain function and neurotransmitter spatial correlations in healthy volunteers: a neuroimaging study

**DOI:** 10.3389/fnhum.2026.1859875

**Published:** 2026-05-29

**Authors:** Yuchun Zheng, Kongqing Li, Gengbiao Zhang, Hongyi Zheng, Jinghua Wu, Wenbin Zheng

**Affiliations:** 1 Department of Radiology, The Second Affiliated Hospital, Shantou University Medical College, Shantou, Guangdong, China; 2 Shantou Longhu District Jinrou Traditional Chinese Medicine Research Institute, Shantou, China; 3 Beijing Jinrou Traditional Chinese Medicine Research Institute, Beijing, China

**Keywords:** ALFF, JuSpace, Meridian-sinew therapy, neurotransmitter spatial correlation, ReHo, resting-state fMRI, working memory

## Abstract

**Objective:**

Non-pharmacological somatic interventions can modulate brain function via body–brain communication, yet the underlying neurobiological mechanisms remain unclear. This study used resting-state fMRI and JuSpace spatial correlation to investigate how meridian-sinew therapy affects regional brain function and its spatial coupling with neurotransmitter systems in healthy volunteers.

**Methods:**

Forty-two healthy volunteers underwent resting-state fMRI and a 2-back working memory task before and after a 50 min meridian-sinew therapy session. We quantified changes in local brain activity using ALFF and ReHo. JuSpace was applied to examine spatial correlations between functional alteration maps and neurotransmitter receptor/transporter density atlases.

**Results:**

Post-intervention reaction time in the 2-back task was significantly reduced. Neuroimaging revealed increased ALFF and ReHo in the prefrontal cortex, and decreased ReHo in the left putamen and insula. Spatial correlation analyses showed that ALFF changes were significantly correlated with 5-HT2a, CB1, mGluR5, DAT, NAT, NMDA, SERT, and VAChT distributions, while ReHo changes correlated only with SERT.

**Conclusion:**

Meridian-sinew therapy modulates regional brain physiology by enhancing prefrontal neural activity and suppressing subcortical interoceptive processing, accompanied by improved working memory efficiency. These functional changes show consistent spatial associations with multiple neurotransmitter systems. Our findings provide multimodal neuroimaging evidence for understanding how somatic interventions shape brain function through body–brain communication pathways.

## 1 Introduction

Rooted in Traditional Chinese Medicine (TCM), meridian-sinew therapy is a non-pharmacological somatic intervention that targets myofascial trigger points (sinew nodules) along meridian-sinew pathways through manual manipulation and scalp acupuncture. This intervention activates somatosensory afferent pathways and modulates connective tissue mechanics, thereby relieving muscular tension and regulating central neural processing (Li and Ma, 2024). Anatomically, meridian-sinew pathways overlap extensively with peripheral nerves projecting to the central nervous system, especially in the cranio-cervical region, providing a neuroanatomical basis for bottom-up body–brain communication (Li and Ma, 2024). Meridian-sinew therapy effectively alleviates neurological conditions such as peripheral facial paralysis, post-stroke sequelae, and cerebral palsy (Zhang et al., 2017; Kang et al., 2024a; Kang et al., 2024b). Moreover, while our prior research demonstrates it improves cognitive function by enhancing cerebral blood flow, regional neural activity, and functional network topology (Gao et al., 2022; Zhang et al., 2024; Zhang et al., 2025a), its central mechanisms remain incompletely understood.

Conventional resting-state fMRI reflects macroscale neural activity but cannot reveal the underlying neurochemical substrates. Regional brain function is tightly regulated by the spatial distribution of neurotransmitter receptors and transporters (Hansen et al., 2022a, 2022b). JuSpace enables spatial correlation analysis between fMRI functional maps and PET/SPECT neurotransmitter atlases (Dukart et al., 2021), offering a novel approach to linking functional changes with neurochemical landscapes. This tool has been successfully applied to reveal molecular underpinnings in various brain disorders (Wang et al., 2024b; Liu et al., 2025).

Against the framework of body–brain communication dynamics, this study combined rs-fMRI (ALFF/ReHo), behavioral assessment, and JuSpace neurochemical mapping to explore how meridian-sinew therapy modulates brain function. We aimed to identify therapy-induced changes in regional intrinsic activity and their spatial associations with neurotransmitter systems, providing multimodal neuroimaging evidence for somatic modulation of brain physiology.

## 2 Materials and methods

### 2.1 Participants

Forty-four right-handed healthy volunteers were recruited. Two were excluded due to excessive head motion. The final sample included 42 participants (18 males, 24 females, mean age 24.76 ± 1.90 years). All had normal brain MRI, no neuropsychiatric disorders, no psychotropic medication use in 3 months, and no recent acupuncture or massage. Additionally, to control for physiological confounds affecting resting-state fMRI, all participants were instructed to ensure adequate sleep (>8 h) the night before the experiment and strictly refrain from consuming alcohol or caffeinated beverages for 24 h prior to the scan. Exclusion criteria included MRI or acupuncture contraindications and head motion >2.0 mm translation or >2.0° rotation. The study was approved by the Ethics Committee of the Second Affiliated Hospital of Shantou University Medical College (No. 2021-19). All participants provided written informed consent.

### 2.2 Experimental design and intervention

A within-subject pre-post design was used. All scans were performed between 14:00 and 18:00 to minimize circadian effects. For the behavioral assessment, working memory was measured using a 2-back task^1^ before and after intervention. Participants practiced until accuracy ≥60%. The intervention consisted of a 50 min Wu Jinghua meridian-sinew therapy (Gao et al., 2022; Zhang et al., 2024, 2025a), comprising 30 min of scalp acupuncture and 20 min of manual sinew-release. Scalp acupuncture utilized sterile disposable needles (0.30 × 50 mm, Huanqiu) at key acupoints, including Baihui (GV20), Sishencong (EX-HN1), and Fengchi (GB20), with an insertion depth of 12.5–20.0 mm. The latter employed a systematic suite of techniques (e.g., pushing, plucking, twisting, kneading, and rubbing) to target the musculature, fascia, and pathological sinew nodules localized along the cervical meridian-sinew pathways. During the scanning phase, resting-state fMRI was acquired before and immediately after interventions.

### 2.3 Instruments and methods

#### 2.3.1 Data acquisition

Scans were performed on a 3.0 T GE MRI scanner equipped with an 8-channel head coil. During the scans, participants were instructed to remain awake, motionless, and with their eyes closed. High-resolution T1-weighted structural images were acquired using a sagittal three-dimensional brain volume imaging (3D-BRAVO) sequence with the following parameters: TR = 5.888 ms, TE = 2.024 ms, slice thickness = 1.0 mm (no gap), FOV = 240 × 240 mm, matrix = 512 × 512, flip angle = 15°, and 176 slices. BOLD-fMRI data were acquired using a gradient-echo echo-planar imaging (GRE-EPI) sequence: TR = 2,000 ms, TE = 30 ms, slice thickness = 3 mm (no gap), FOV = 240 × 240 mm, matrix = 64 × 64, flip angle = 90°, 33 slices, and 210 volumes (total scan time: 7 min).

#### 2.3.2 Data preprocessing and metric calculation

Preprocessing was performed using the RESTplus toolbox^2^ on MATLAB R2020a. Initial procedures included discarding the first 10 volumes, slice-timing correction, head-motion correction, and T1-based spatial normalization to the MNI space (resampled to 3 × 3 × 3 mm^3^). The pipelines then diverged for metric calculation. For ALFF, the normalized data were spatially smoothed with a 6 mm full-width at half-maximum (FWHM) Gaussian kernel, followed by detrending, nuisance regression, and band-pass filtering (0.01–0.08 Hz) before computation. For ReHo, the normalized data directly underwent detrending, nuisance regression, and band-pass filtering. To avoid artificially inflating regional homogeneity, ReHo was calculated on these non-smoothed data, and the resulting maps were subsequently smoothed with the same 6 mm kernel. In both pipelines, nuisance regression utilized the rigorous Friston 24-parameter model alongside the explicit regression of cerebrospinal fluid and white matter signals to mitigate physiological noise.

### 2.4 Statistical analysis

Analyses were conducted using SPSS 26.0 and MATLAB R2020a. For the fMRI data, we used paired-sample *t*-tests to compare pre- and post-intervention ALFF and ReHo maps, applying Gaussian Random Field (GRF) correction (voxel *p* < 0.001, cluster *p* < 0.05). For behavioral data, mean reaction times (excluding incorrect responses, values <100 ms, and values >3 standard deviations from the mean) and accuracies were analyzed using paired-sample *t*-tests (*p* < 0.05). Pearson correlation analysis, corrected via False Discovery Rate (FDR), was used to explore relationships between functional and behavioral changes.

To investigate potential neurochemical mechanisms, spatial correlation analyses were performed using the JuSpace toolbox (v1.5, https://github.com/juryxy/JuSpace) by incorporating PET/SPECT density atlases for 15 neurotransmitter receptors and transporters (D1, D2, DAT, FDOPA, 5-HT1a, 1b, 2a, 4, SERT, NMDA, GABAa, NAT, mGluR5, VACHT, and CB1). These neurochemical maps were selected as they represent standardized and validated atlases within the JuSpace framework, encompassing the primary excitatory, inhibitory, and modulatory systems essential for somatic and cognitive function. Following the resampling of unthresholded functional difference maps to ensure spatial consistency with these PET/SPECT templates, regional average t-values and neurotransmitter densities were extracted based on the default Neuromorphometrics atlas (comprising 119 cortical and subcortical regions) to compute spatial Spearman correlation coefficients. To rigorously account for the inherent spatial autocorrelation of neuroimaging data, statistical significance was established via exact permutation testing (spin tests, 10,000 permutations), with a subsequent FDR correction applied for multiple comparisons.

## 3 Results

### 3.1 Behavioral results

As shown in Table 1, participants demonstrated a significant decrease in mean reaction time during the 2-back task post-intervention (*p* < 0.05). Conversely, task accuracy remained stable between pre- and post-intervention assessments (*p* > 0.05). This absence of significant improvement in accuracy was likely driven by a ceiling effect, given the participants’ already high baseline performance.

**Table 1:** Comparison of 2-back task performance before and after intervention (mean ± SD).

Variables	Pre-intervention	Post-intervention	*p*-value
2-back accuracy (%)	90.40 ± 7.28	91.96 ± 6.55	0.252
2-back reaction time (s)	0.72 ± 0.16	0.67 ± 0.20	0.035^*^

*p < 0.05.

### 3.2 Regional brain function alterations

**Figure 1:**
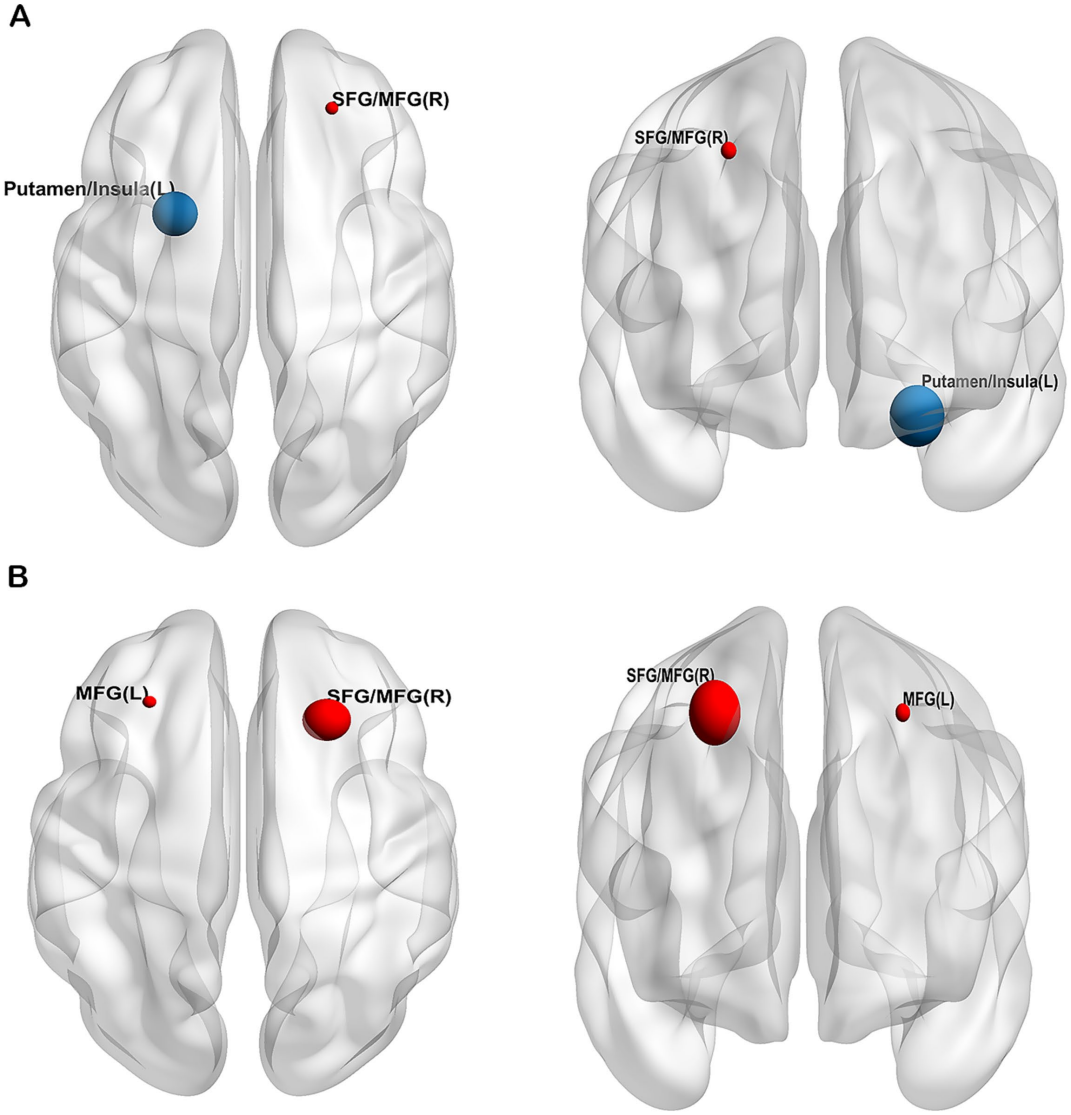
Brain regions showing altered regional function pre- and post-intervention. **(A)** Significant changes in regional homogeneity (ReHo). **(B)** Significant changes in the amplitude of low-frequency fluctuations (ALFF). Red clusters indicate increased metrics, whereas blue clusters indicate decreased metrics. SFG, superior frontal gyrus; MFG, middle frontal gyrus; L, left; R, right.

Compared to baseline, ReHo was significantly decreased in the left putamen and left insula, accompanied by a significant increase in the right superior and middle frontal gyri (voxel *p* < 0.001, cluster *p* < 0.05, GRF corrected; Figure 1A, Table 2).

**Table 2:** Brain regions showing significant differences in ReHo and ALFF between pre- and post-intervention.

Cluster	Regions	Peak MNI (*x*, *y*, *z*)	Peak *t*-value	Voxels	Mean value (pre vs. post)
ReHo					
1	Putamen (L)	−24, 6, −15	−4.91	27	1.14 ± 0.08 vs. 1.07 ± 0.09
	Insula (L)			20	
2	SFG (R)	24, 39, 45	4.76	46	1.08 ± 0.10 vs. 1.15 ± 0.10
	MFG (R)			10	
ALFF					
1	SFG (R)	24, 27, 48	4.8	39	1.22 ± 0.24 vs. 1.40 ± 0.32
	MFG (R)			20	
2	MFG (L)	−27, 33, 48	4.89	24	1.37 ± 0.44 vs. 1.56 ± 0.43

ReHo, regional homogeneity; ALFF, amplitude of low-frequency fluctuation; MNI, Montreal Neurological Institute space coordinates; L, left; R, right; SFG, superior frontal gyrus; MFG, middle frontal gyrus.

Similarly, we observed a significant post-intervention increase in ALFF within the right superior and bilateral middle frontal gyri (GRF corrected; Figure 1B, Table 2). Notably, no brain regions exhibited a significant decrease in ALFF.

### 3.3 Brain-behavior correlations

In exploratory, uncorrected correlation analyses, we observed a positive association between intervention-induced ALFF changes in the left middle frontal gyrus and changes in 2-back task accuracy (*r* = 0.318, *p* = 0.040). However, this correlation did not survive FDR correction for multiple comparisons across all tested brain-behavior pairs and should therefore be interpreted with caution as a preliminary observation requiring replication. No significant correlations were found between changes in mean 2-back reaction times and ALFF or ReHo alterations in any identified regions.

### 3.4 Neurotransmitter spatial correlations

**Figure 2:**
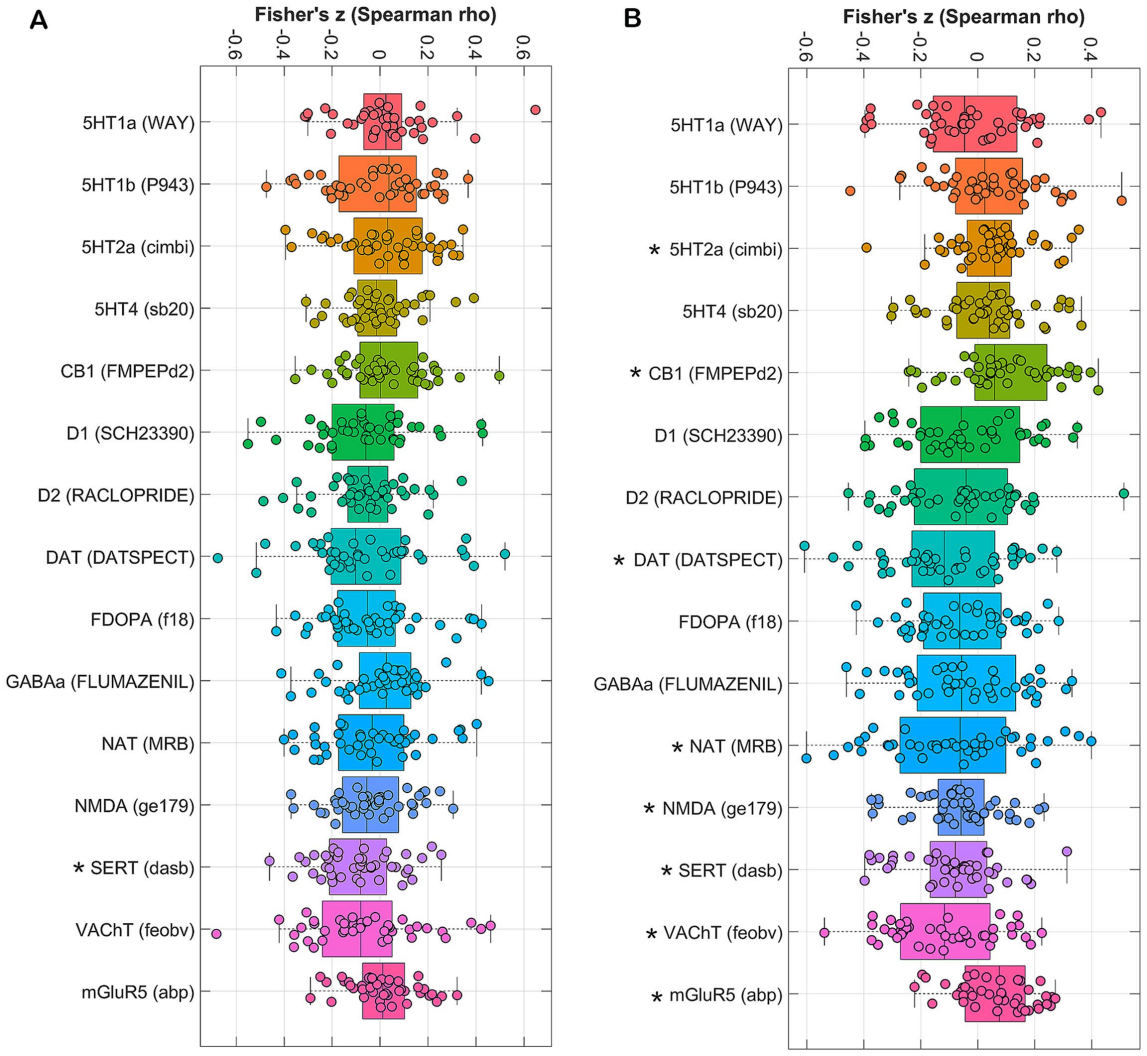
Spatial correlations between therapy-induced functional alterations and neurotransmitter systems. **(A)** Spatial correlations between ReHo maps and the distribution of neurotransmitter receptors and transporters. **(B)** Spatial correlations between ALFF maps and the distribution of neurotransmitter receptors and transporters. Asterisks (*) indicate significant correlations after false discovery rate (FDR) correction (*p* < 0.05). ReHo, regional homogeneity; ALFF, amplitude of low-frequency fluctuations; 5-HT, serotonin; CB1, cannabinoid receptor type 1; D1/D2, dopamine receptors; DAT, dopamine transporter; FDOPA, 6-fluorodopa; GABAa, gamma-aminobutyric acid type A; NAT, noradrenaline transporter; NMDA, N-methyl-D-aspartate; SERT, serotonin transporter; VAChT, vesicular acetylcholine transporter; mGluR5, metabotropic glutamate receptor 5.

Spatial correlation analysis demonstrated that therapy-induced ALFF alterations were significantly correlated with the density maps of multiple neurotransmitter receptors and transporters (all FDR corrected). Specifically, ALFF changes showed positive correlations with the distributions of 5-HT2A (*r* = 0.06, *p* = 0.025), CB1 (*r* = 0.09, *p* = 0.002), and mGluR5 (*r* = 0.06, *p* = 0.008), whereas we observed significant negative correlations with DAT (*r* = −0.11, *p* = 0.002), NAT (*r* = −0.09, *p* = 0.041), NMDA (*r* = −0.07, *p* = 0.010), SERT (*r* = −0.09, *p* = 0.002), and VAChT (*r* = −0.12, *p* = 0.002) (Figure 2B). In contrast, ReHo changes exhibited a significant negative spatial correlation solely with SERT (*r* = −0.09, *p* = 0.006, FDR corrected; Figure 2A).

## 4 Discussion

This study demonstrates that meridian-sinew therapy modulates regional brain function and improves working memory efficiency in healthy adults, with consistent spatial associations across multiple neurotransmitter systems. These findings provide novel insights into the central neuromodulatory mechanisms of meridian-sinew therapy from functional and neuroimaging perspectives by employing rs-fMRI and JuSpace-based spatial correlation analysis.

### 4.1 Meridian-sinew therapy optimizes brain function via neural modulation

Our findings indicate that meridian-sinew therapy enhanced ALFF and ReHo in the prefrontal cortex, a core region for working memory and executive control. Concurrently, ReHo was reduced in the putamen and insula, key nodes of the salience and interoceptive networks. These changes suggest that peripheral somatosensory input from the therapy generates bottom-up signals that reconfigure resting-state brain activity: enhancing task-relevant prefrontal resources while suppressing non-essential interoceptive processing. The middle frontal gyrus serves as a critical working memory processing node within the frontoparietal network, a role well-supported by the Parieto-Frontal Integration Theory (P-FIT) model (Jung and Haier, 2007). A meta-analysis by Owen et al. (2005) confirmed the bilateral prefrontal cortex as a core region consistently activated during N-back tasks. Additionally, prior studies show that external TCM interventions, like acupuncture and moxibustion, exert central regulatory effects by modulating activity and connectivity in cognition-related areas, particularly the prefrontal cortex (Cai et al., 2018; Zheng et al., 2018; Lai et al., 2022). As a comparable non-pharmacological physical intervention, meridian-sinew therapy might modulate cognition-related brain regions through potentially similar central modulatory mechanisms.

Consequently, the enhanced resting-state activity observed in the bilateral prefrontal cortex in this study is less likely to be a mere inefficient compensatory response. Instead, it could plausibly reflect a “neural priming effect” induced by meridian-sinew therapy. Potentially driven by the bottom-up transmission of somatosensory stimuli, this effect may shift the executive control network into a preparatory state with increased reserves, facilitating its recruitment during subsequent cognitive tasks. Mechanistically, somatosensory feedback from the therapy may adjust cortical excitability by altering resting membrane potentials, a process analogous to the mechanisms underlying transcranial direct current stimulation (tDCS) (Dadgar et al., 2022). This shift might recalibrate neural response thresholds and thereby potentially improve the efficiency of brain resource allocation.

Interestingly, ReHo significantly decreased in the left putamen and insula, despite no distinct ALFF changes. As a core node of the salience network, the insula collaborates with the putamen to process interoceptive and emotional information. This reduction in ReHo may reflect the suppression of non-task-related stimuli, such as interoceptive signals from the putamen and insula (Fermin et al., 2024), suggesting optimized resting-state resource allocation. As Neubauer and Fink (2009) noted in their revised hypothesis, highly cognitively efficient individuals must fully recruit prefrontal resources under high cognitive load to ensure processing accuracy. Therefore, by reshaping resting-state functional states, meridian-sinew therapy may help establish an optimal processing environment characterized by higher neural capacity and an improved signal-to-noise ratio. This could facilitate a more efficient allocation of cognitive resources during complex challenges like the 2-back task, ultimately contributing to improved cognitive efficiency.

### 4.2 Neurochemical substrates underlying the neuromodulatory effects of Meridian-sinew therapy

A key advantage of non-pharmacological interventions is their capacity to engage multiple neurobiological systems through naturalistic physiological pathways, rather than targeting a single receptor. Consistent with this, acupuncture modulates specific regional brain functions via diverse neurotransmitter systems (Wang et al., 2024a). Furthermore, neuroimaging evidence indicates that acupuncture-induced brain network activation patterns spatially overlap with specific receptor distributions (Zhang et al., 2025b). As a comparable physical TCM therapy, meridian-sinew therapy might share similar modulatory mechanisms. To explore the neurochemical context of the functional changes observed in this study, we employed JuSpace spatial correlation analysis. JuSpace analyses revealed that functional changes were spatially coupled with serotonergic, endocannabinoid, dopaminergic, noradrenergic, cholinergic, and glutamatergic systems. Unlike single-target pharmacological agents, meridian-sinew therapy engages a broad neurochemical landscape, a hallmark of physiological body–brain communication.

First, a potential synergy between the endocannabinoid and serotonin systems could help explain the overall relaxation and emotional-cognitive regulatory effects of the therapy. Our findings show that ALFF alterations positively couple with CB1 and 5-HT2a distributions while negatively correlating with SERT. Given that the CB1 system is a pivotal regulator of synaptic plasticity and systemic equilibrium (Busquets-Garcia et al., 2022; Gao and Asim, 2025), this coupling suggests that meridian-sinew therapy may preferentially modulate CB1-enriched brain regions, potentially providing a structural basis for adaptive remodeling to restore physiological homeostasis. Concurrently, the correlation patterns with 5-HT2a and SERT indicate that functional alterations likely involve regions with dense 5-HT2a expression and prolonged serotonin clearance, such as the middle temporal lobe and prefrontal cortex (Beliveau et al., 2017). Together, these features suggest a functional convergence: CB1-mediated stabilization may optimize the neurochemical environment, thereby facilitating prefrontal-dependent processes, such as working memory, while the serotonergic system refines signal processing within these cognitive circuits.

Second, a shared pattern of spatial alignment with signal clearance between monoaminergic and cholinergic systems points to their potentially synergistic role in optimizing higher-order cognition. ALFF changes negatively correlated with the distributions of DAT, NAT, and VAChT, indicating that therapy-induced regional brain function alterations tend to occur in areas with slower neurotransmitter clearance. This spatial overlap with low-clearance regions could theoretically provide an anatomical substrate for prolonged monoamine and acetylcholine signaling, thereby potentially amplifying their neuromodulatory efficacy. Specifically, such prolonged signaling may fine-tune catecholaminergic homeostasis within the prefrontal cortex, effectively exploiting the inverted-U principle to shift prefrontal activity from a suboptimal state toward an optimal operating range. This optimization is thought to enhance advanced cognitive functions, including working memory, attentional control, and decision-making (Li et al., 2023). Similarly, lower VAChT density might provide a physical substrate for sustained cholinergic signaling, which regulates baseline arousal and potentially optimizes the preparatory state and signal transmission of cortical circuits (Parikh and Bangasser, 2020). Thus, through this shared spatial association with low-clearance mechanism, these systems might work together to enhance neural processing tied to higher cognition.

Furthermore, potential antagonism and compensation among glutamatergic receptor subtypes could represent a synergistic mechanism that maintains homeostasis and dynamic flexibility. ALFF changes negatively correlated with NMDA distribution but positively correlated with mGluR5. Opposite correlations with NMDA and mGluR5 indicate balanced excitatory plasticity and dynamic flexibility. Given that NMDA is a critical mediator of hippocampal long-term potentiation (Ramirez-Exposito et al., 2026), this negative correlation does not necessarily indicate functional inhibition. Instead, the therapy predominantly targets core synaptic plasticity regions to foster a low-baseline homeostasis, thereby optimizing the neural signal-to-noise ratio. Conversely, the positive correlation with mGluR5 suggests a compensatory mechanism, wherein functional shifts in mGluR5-rich areas modulate excitatory thresholds to offset cognitive flexibility deficits induced by NMDA fluctuations (Hagena and Manahan-Vaughan, 2022). Therefore, a hypothesized synergy between NMDA-mediated core homeostasis and mGluR5-dependent dynamic gain may prime brain networks during the resting state, possibly enabling rapid and flexible transitions to highly efficient cognitive states when demanded.

Finally, ReHo analysis highlighted a more localized modulatory pattern. Unlike the broad correlations observed with ALFF, ReHo changes primarily exhibited a significant negative correlation with SERT, suggesting a potential mechanism in which the spatial distribution of serotonin clearance dynamics may anatomically align with local neural synchronization. We propose that regions with high SERT distribution (e.g., the insula) promote desynchronization via rapid signal clearance, whereas low-SERT regions (e.g., the prefrontal cortex) facilitate signal focusing and synchronization (Beliveau et al., 2017). This proposed optimization of the local signal-to-noise ratio might serve as a key molecular mechanism by which meridian-sinew therapy modulates regional neural synchronization and redistributes neural resources.

Collectively, the JuSpace results reveal that the functional effects of this non-pharmacological intervention are not randomly distributed across the brain but show a coherent spatial alignment with the topology of multiple neurotransmitter systems. The convergence of associations across serotonergic, endocannabinoid, monoaminergic, cholinergic, and glutamatergic systems suggests that the therapy’s effects on brain physiology may involve a multi-system neurochemical landscape. This multi-system engagement distinguishes non-pharmacological somatic interventions from pharmacological agents that typically target individual receptor systems, and may underlie their broader, more integrative effects on brain function and cognition.

These results indicate that meridian-sinew therapy reshapes brain function through a coordinated multi-neurotransmitter system, supporting the integrative nature of body–brain communication.

### 4.3 Implications for the central neuromodulatory effects of meridian-sinew therapy

Our study proposes a mechanistic model in which peripheral somatic stimulation initiates afferent signaling, subsequently driving prefrontal activation and subcortical modulation. These neural changes modulate neurochemical network coupling, ultimately culminating in improved cognitive efficiency. This framework aligns with the dynamics of body–brain communication and underscores non-pharmacological somatic therapies as a promising strategy for regulating brain function.

## 5 Limitations and future directions

Certain limitations of this study warrant consideration. First, the relatively small sample size may constrain the statistical power and generalizability of the findings; consequently, future research should prioritize large-scale, multi-center cohorts to enhance the stability and reliability of these results. Second, the absence of additional experimental groups with varying intervention durations and frequencies limits the characterization of how these parameters modulate dynamic brain function. Subsequent studies should establish multiple intervention gradients to thoroughly explore the dose–response relationship of meridian-sinew therapy. Third, regarding behavioral outcomes, although a pre-experiment practice session with a strict accuracy threshold was implemented to minimize practice effects, the shortened reaction times observed post-intervention may still be influenced by residual short-term procedural memory and strategy optimization. As these confounders are inherent to single-group within-subject designs, future studies should incorporate a sham intervention or a passive control group to effectively isolate the therapeutic impact from such practice effects. Furthermore, the lack of improvement in task accuracy is likely attributable to a ceiling effect. To more sensitively capture cognitive enhancements, future research should implement multidimensional assessments encompassing broader cognitive domains, such as sustained attention and emotional regulation, utilizing more demanding paradigms like 3-back or adaptive n-back tasks to provide a more comprehensive evaluation of intervention-induced cognitive plasticity. Finally, the analysis of receptor and transporter distribution remains primarily correlational and does not directly confirm real-time neurochemical fluctuations. To validate these findings, future studies would benefit from multimodal imaging, such as simultaneous PET-MRI, or *in vivo* animal models.

## 6 Conclusion

Meridian-sinew therapy modulates regional intrinsic brain activity by enhancing prefrontal function and reducing subcortical interoceptive synchronization, accompanied by improved working memory. These changes show spatial correlations with multiple neurotransmitter systems. This study provides multimodal neuroimaging evidence for body–brain communication and reveals how non-pharmacological somatic interventions regulate human brain function.

## Data Availability

The raw data supporting the conclusions of this article will be made available by the authors, without undue reservation.

## Footnotes

1
https://experiment.psych.ac.cn/

2
https://restfmri.net/forum/RESTplus
